# Regulatory Action of Calcium and Calcium Channels in Pain Pathways

**DOI:** 10.7150/ijbs.110504

**Published:** 2025-05-31

**Authors:** Yan Zhang, John Shannonhouse, Hyeonwi Son, Joon Tae Park, Yu Shin Kim

**Affiliations:** 1 Department of Oral and Maxillofacial Surgery, University of Texas Health Science Center at San Antonio, San Antonio, TX, USA.; 2 Programs in Integrated Biomedical Sciences, Translational Sciences, Biomedical Engineering, Radiological Sciences, University of Texas Health Science Center at San Antonio, San Antonio, TX, USA.; 3 Division of Life Sciences, College of Life Sciences and Bioengineering, Incheon National University, Incheon, South Korea.

**Keywords:** Ca^2+^, Ca^2+^ channel, VGCC, TRP channel, GCaMP, GECI, pain, regulatory mechanism

## Abstract

Calcium ions (Ca^2+^) and Ca^2+^ channels are pivotal in the regulation of pain pathways and serve as key regulators of neuronal excitability and neurotransmitter release. We review the different types of Ca^2+^ channels involved in pain processing, including voltage-gated Ca^2+^ channels (VGCCs), such as L-, N-, P/Q-, and T-type channels. Each subtype is intricately involved in different aspects of pain perception, from acute pain signaling to the development and maintenance of chronic pain states. In addition, the roles of transient receptor potential (TRP) channels, particularly TRPV1 and TRPA1, are discussed in the context of their contribution to chronic pain. Advances in Ca^2+^ imaging techniques, particularly through genetically encoded Ca^2+^ indicators (GECIs), such as GCaMPs, have provided unprecedented insight into the dynamic role of Ca^2+^ channels in pain pathways. These efforts have deepened our understanding of Ca^2+^ channels and suggest novel therapeutic targets for more effective pain management strategies within Ca^2+^ channels.

## Introduction

Pain is an important biological response that alerts the body to potential injury or harm. protects the body from injury or potential harm. Acute pain is usually temporary, but chronic pain may persist or recur for more than three months, often resulting in long-term physical and psychological problems 1. At the forefront of pain detection are specialized sensory neurons called nociceptors, which act as primary sensors for noxious stimuli 2. These cells initiate the complex pain process that ultimately leads to the conscious perception of pain. Central to this intricate system are Ca^2+^ channels, which play a pivotal role in pain signaling.

Ca^2+^ channels are essential components of the pain signaling apparatus, significantly affecting the transmission and perception of nociceptive stimuli 3. Ca^2+^ channels are widely present throughout the nervous system and play a key role in regulating pain pathways 3. Specifically, Ca^2+^ channels regulate neurotransmitter release, neuronal excitability, and signal transmission in the pain pathway. In peripheral nociceptors, Ca^2+^ channels contribute to the detection and initial encoding of noxious stimuli. In the dorsal root ganglia, they modulate the excitability of sensory neurons and influence pain signal processing. In the central terminals of the spinal cord, Ca^2+^ channels are important for neurotransmitter release and synaptic transmission of pain signals to second-order neurons. These diverse roles of Ca^2+^ channels in pain signaling make them particularly attractive targets for pain therapeutic strategies.

*In vivo* Ca^2+^ imaging techniques have been developed and have become invaluable tools for exploring the role of Ca^2+^ and Ca^2+^ channels in pain pathways. By allowing real-time observation of Ca^2+^ dynamics in living organisms, Ca^2+^ imaging provides insight into how Ca^2+^ and Ca^2+^ channels function within intact neural circuits during pain processing. These techniques also allow for direct observation of the role in regulating neuronal excitability and neurotransmitter release by visualizing the activation and modulation of Ca^2+^ in response to nociceptive stimuli. The ability to observe these processes in real-time is essential for identifying the specific contributions of different Ca^2+^ channel subtypes to pain signals and developing targeted therapeutic interventions. To date, *in vivo* Ca^2+^ imaging has been used effectively to study neuronal and non-neuronal cell populations in the brain 4, 5, spinal cord 6, 7, and primary sensory neurons 8, 9 in living mice. It also has been applied to a variety of pain models 10-12, providing a powerful tool for investigating the complex processes underlying pain perception and other sensory functions.

We discuss the regulatory role of Ca^2+^ and Ca^2+^ channels in pain pathways, highlighting how visualization of Ca^2+^ imaging has improved our understanding of these mechanisms. We examine the specific roles of Ca^2+^ channels in pain transmission and modulation, with the aim of elucidating their potential as therapeutic targets for the management of chronic pain. We also describe the critical role of advanced imaging techniques in unraveling the complex intricacies of pain biology, thereby providing insights that may lead to more effective treatments for chronic pain conditions.

## Types of Ca^2+^ Channels in Pain Pathways

### Voltage-Gated Ca^2+^ Channels (VGCCs)

**Figure 1:**
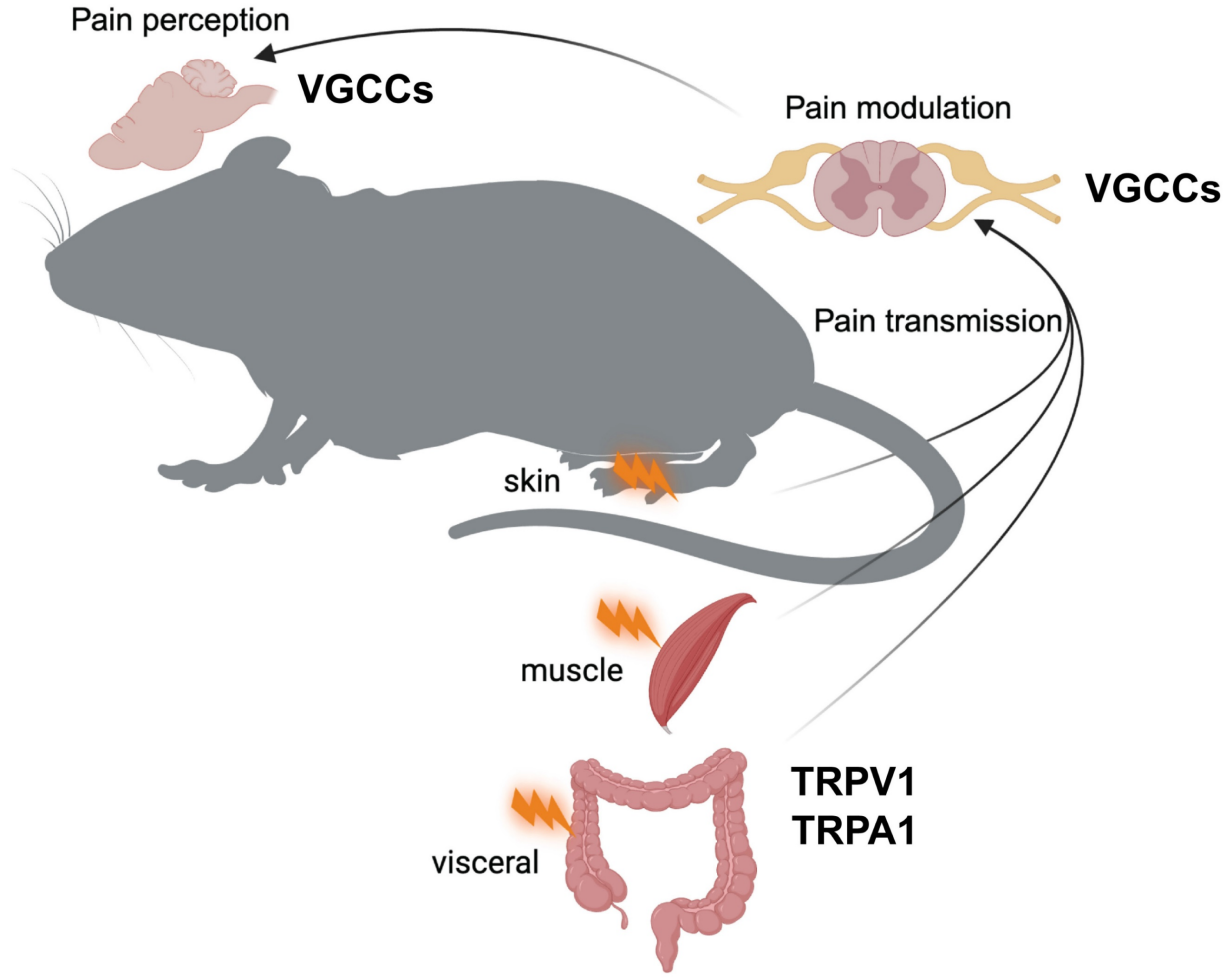
Depiction of the journey of pain signals from peripheral tissues to the brain, where they are ultimately perceived as pain. Pain signals are initiated in specialized sensory nerve endings called nociceptors, located in the skin, muscles, and organs (e.g., visceral tissues). In these nociceptors, Ca²⁺-permeable channels, TRPV1 and TRPA1, play a key role in detecting noxious stimuli, such as mechanical injury, temperature extremes (hot or cold), and chemical irritants. When these channels are activated, pain signals are initiated. Transmission of these pain signals to the central nervous system is facilitated by voltage-gated Ca²⁺ channels (VGCCs) in the presynaptic terminals of the spinal cord, which trigger the release of neurotransmitters and allow the continuation of the pain signal through the ascending pathways. VGCCs play a key role in pain modulation by affecting the intensity and duration of pain signals within the spinal cord and higher brain regions, ultimately influencing the perception and experience of pain.

Neurons typically express several types of voltage-gated Ca^2+^ channels (VGCCs), which are classified as high-voltage-activated (HVA) and low-voltage-activated (LVA) channels based on their activation thresholds 13. HVA channels require stronger depolarization to open and are typically activated at potentials more positive than -40 mV. HVA channels include L-type (Cav1.1-Cav1.4), P/Q-type (Cav2.1), N-type (Cav2.2), and R-type (Cav2.3) channels, each of which provide unique contributions to pain processing 14. HVA channels are pharmacologically distinct and consist of heteromultimeric complexes containing a pore-forming α1 subunit, one of four types of β subunits, and one of four types of α2δ subunits 15. In contrast, LVA channels, also known as T-type channels, are activated at more negative membrane potentials (approximately -70 to -60 mV). LVA channels include Cav3.1, Cav3.2, and Cav3.3. Unlike their HVA counterparts, LVA channels appear to function as monomers composed solely of α1 subunits. The functional importance of HVA and LVA channels in pain signal transduction becomes apparent at the synaptic level. When an action potential reaches the presynaptic terminal of a nociceptive neuron, membrane depolarization occurs. This depolarization opens VGCCs, allowing Ca^2+^ ions to enter the cell. The resulting influx of Ca^2+^ ions causes the release of neurotransmitters such as glutamate, calcitonin gene-related peptide (CGRP), and substance P into the synaptic cleft. These neurotransmitters then bind to receptors on the postsynaptic neuron, propagating the pain signal (Figure 1).

HVA Ca^2+^ channels depend critically on their auxiliary α2δ subunit, which orchestrates multiple aspects of channel function despite not participating directly in ion conduction. Encoded by one of four genes (*CACNA2D1-4*) 16, with α2δ1 being the most prevalent in pain-processing neurons 17, this glycosylated subunit profoundly modulates the α1 subunit. The α2δ subunit fine-tunes Ca^2+^ channel biophysics by enhancing opening probability, shifting voltage-dependent activation toward more negative potentials, and accelerating both activation and inactivation kinetics 18, 19. Beyond these electrophysiological effects, α2δ governs channel trafficking and membrane stabilization, facilitating movement from the endoplasmic reticulum to the plasma membrane and extending channel residence time at the cell surface 16, 20, 21. In pain signaling, the α2δ1 subunit plays a crucial role, particularly in pathological conditions such as neuropathic pain 17. Following nerve injury, α2δ1 expression undergoes marked upregulation in affected neural circuits 22, 23. This molecular adaptation enhances calcium influx through both Cav2.2 and Cav1.2 channels in dorsal root ganglia neurons and their central projections in the spinal dorsal horn 24-26. The resulting amplification of excitatory neurotransmitter release drives central sensitization, a hallmark of chronic pain.

#### L-type (Cav1.2 and Cav1.3)

Among the four subtypes of HVA channels, only L-type Cav1.2 and Cav1.3 channels are predominantly found in neurons, including medium-sized dorsal root ganglia (DRG) neurons as well as the cell bodies and dendrites of dorsal horn neurons 27. Cav1.2 and Cav1.3 have distinct roles in pain transmission and the development of neuropathic pain. In the spinal dorsal horn, Cav1.3 channels are primarily involved in short-term sensitization during physiological pain transmission, whereas Cav1.2 channels play a key role in long-term plasticity associated with neuropathic pain 28, 29. Peripheral nerve injury results in the accumulation of VGCC auxiliary units in primary afferent terminals, which increases the activity of Cav1.2 channels in the spinal dorsal horn and contributes to the maintenance of the neuropathic pain state 24. Cav1.2 channels are also found in peripheral afferent neurons 30, but their role at this location is not considered significant in the development of neuropathic pain. Pharmacological interventions targeting these channels have shown nuanced results. L-channel blockers such as nitrendipine have been shown to reduce hyperalgesia (increased sensitivity to pain) and mechanical allodynia (pain evoked by normally non-painful stimuli) in animal models of neuropathic pain but are generally not considered as effective or well established as other Ca^2+^ channel modulators, such as gabapentin and pregabalin, which bind to the α2δ subunit for the management of neuropathic pain.

#### N-type (Cav2.2)

Cav2.2 channels are abundantly expressed in key nociceptive structures, including dorsal horn neurons and DRG neurons 3. At the cellular level, Cav2.2 channels control Ca^2+^ influx at presynaptic terminals, thereby regulating the release of neurotransmitters involved in pain signaling 31. Interestingly, Cav2.2 channels in peripheral nociceptors have been suggested to be required for thermal hypersensitivity but not for intradermal capsaicin-induced mechanical hypersensitivity 32. During inflammation induced by complete Freund's adjuvant (CFA), Cav2.2 channels have been found to be upregulated in thermal-sensitive DRG neurons but not in mechanical-sensitive DRG neurons, and blocking Cav2.2 channels inhibited CFA-induced thermal hyperalgesia but not mechanical hyperalgesia 33. These results suggest that Cav2.2 channels play a critical role in regulating thermal pain responses during acute and chronic inflammation. The importance of Cav2.2 channels in pain processing has been further elucidated by genetic studies. Mice deficient in N-type channels demonstrate reduced nociceptive responses and attenuated inflammatory and neuropathic pain-related behaviors 34-36, ​​confirming the important role of Cav2.2 channels in nociceptive processing. Therefore, Cav2.2 channels are considered an important therapeutic target for pain management. Selective inhibition of these channels has shown promise in reducing pain responses in a variety of animal models. A notable example is the Cav2.2-specific peptide pore blocker ziconotide (Prialt™). Ziconotide is a synthetic version of the ω-conotoxin MVIIA and is clinically approved for the treatment of refractory chronic pain 37. However, the clinical application of ziconotide is limited by the need for intrathecal administration and potential side effects, which limit its widespread use. Research efforts are underway to develop novel modulators of Cav2.2 channels. For example, the peptidomimetic compound CBD3063 has been shown to selectively modulate Cav2.2 activity and reduce pain behaviors without significant side effects 38. Peptidomimetic modulators targeting the interaction of collapsin response mediator protein 2 (CRMP2) with Cav2.2 channels have shown promise in alleviating chronic pain in preclinical models and in reversing nociceptive behaviors in rodents without eliciting sensory, affective, or cognitive effects 39. These results highlight the potential of targeted therapies that can modulate Cav2.2 function to achieve analgesia.

#### P/Q-type (Cav2.1)

P/Q-type Ca^2+^ channels are found primarily at presynaptic terminals in the dorsal horn; their distribution pattern is complementary to that of N-type channels 40. Although their precise role in pain processing is not fully understood, P/Q-type channels are thought to modulate synaptic transmission, particularly in the context of postsynaptic nociceptive transmission. In terms of pain modulation, the involvement of P/Q-type channels appears to vary depending on the type of pain. For instance, in neuropathic pain models, blocking P/Q-type channels with ω-agatoxin IVA does not significantly affect mechanical allodynia 41. However, studies in mice lacking the Cav2.1 α1 subunit have revealed a dual role for P/Q-type channels in pain modulation. They contribute to reducing sensitivity to non-injurious noxious thermal stimuli (antinociceptive role), while promoting pain sensitivity in inflammatory and neuropathic states (pronociceptive role) 42. Mutations in the *CACNA1A* gene, which encodes the Cav2.1 α1 subunit, have been found in patients with trigeminal neuralgia, a debilitating facial pain disorder 43. These mutations have also been associated with familial hemiplegic migraine, a severe form of migraine with aura 44. Mice carrying gain-of-function mutations in this gene display a phenotype associated with trigeminal pain, providing a valuable model to study the genetic and physiological mechanisms underlying trigeminal neuralgia and familial hemiplegic migraine.

#### T-type (Cav3.1, Cav3.2, Cav3.3)

T-type Ca^2+^ channels exhibit a unique structural composition that sets them apart from HVA Ca^2+^ channels. While HVA channels are hetero-multimers composed of a pore-forming α1 subunit and auxiliary subunits (β, α2δ, γ), T-type channels are monomers composed solely of the α1 subunit 45. This structural simplicity has profound implications for their function and regulation. The absence of auxiliary subunits in T-type channels contributes to their distinctive electrophysiological profile characterized by low-voltage activation and fast deactivation kinetics. These properties may contribute to important roles in neural functions, including generation of low-threshold Ca^2+^ spikes, facilitation of burst firing in diverse neuronal populations, and regulation of neurotransmitter release at dorsal horn synapses. T-type Ca^2+^ channels are also highly expressed in both peripheral nociceptive neurons and dorsal horn neurons 46-48. *In vitro* electrophysiological recordings have shown that genetic deletion or pharmacological inhibition of these channels results in diminished action potential firing 49-51, highlighting the importance of these channels in neuronal excitability. Cav3.2 channels, a subtype of T-type Ca^2+^ channels, play an important role in mechanosensation and pain perception. In mechanosensation, Cav3.2 channels serve as selective markers for Aδ-LTMRs and C-LTMRs, two major types of low-threshold mechanoreceptors that innervate skin hair follicles 52. Cav3.2 regulates light-touch perception and nociceptive sensation and is essential for the development of allodynia symptoms in neuropathic pain. Upregulation of Cav3.2 channels has been implicated in a variety of chronic pain conditions, including inflammatory pain 53-55, neuropathic pain (diabetes-induced 56, 57 and nerve injury-induced 58-62), visceral pain 63, 64, bone cancer pain 65, and chemotherapy-induced peripheral neuropathy (e.g., paclitaxel-induced hypersensitivity 66). In the trigeminal system, Cav3.2 channels can impact pain transmission and contribute to trigeminal-mediated neuropathic pain 67-69. Mutations in the *CACNA1H* gene, which encodes the Cav3.2 α1 subunit, have been found in patients with trigeminal neuralgia. These mutations may alter the biophysical properties of these channels 70, 71 and potentially contribute to the pathophysiology of the condition.

Modulation of Cav3.2 channels offers a variety of avenues for therapeutic intervention for pain management, a particular focus of which has been the development of specific Cav3.2 channel blockers. The specific T-type Ca^2+^ channel blocker, TTA-P2, has been shown to reduce hypersensitivity in animal models of diabetic neuropathic pain 72, osteoarthritis pain 73, spinal cord injury-induced neuropathic pain 74, nerve-injury induced pain 75 and inflammatory pain 72. Z944, a highly selective piperazine-based T-type channel blocker, has shown effectiveness in reducing pain behaviors in multiple rodent pain models, including neuropathic pain 76, inflammatory pain 77 and trigeminal pain 70. Despite the promising preclinical results, no T-type Ca^2+^ blockers are currently available on the market for pain management.

Targeting posttranslational modifications such as glycosylation, phosphorylation, and ubiquitination offers promising avenues for developing novel pain management therapies by modulating T-type channel function and expression. Glycosylation of CaV3.2 channels involves the addition of carbohydrate groups (N-linked glycan chains) to asparagine residues on the channel protein, which can influence the channel's surface expression, stability, and gating properties 78, 79. In animal models of painful peripheral diabetic neuropathy in both type 1 and type 2 diabetes, glycosylation of Cav3.2 channels in nociceptors contributes to increased neuronal excitability 80-82. The increased presence and activity of these channels on the cell surface amplifies pain signaling, leading to pain hypersensitivity. *In vitro*, inhibition of the glycosylation process by de-glycosylating enzymes such as neuraminidase or PNGase-F reduces T-type current density in nociceptors and recombinant Cav3.2 channels, as shown in electrophysiological recordings 80, 81. *In vivo*, selective reversal of Cav3.2 channel glycosylation can alleviate pain hypersensitivity in diabetic models 80-83, highlighting the importance of glycosylation in modulating Cav3.2 function and its role in diabetic pain pathways. Phosphorylation is another important and dynamic regulatory mechanism for Cav3.2 channels. Phosphorylation targets the intracellular links connecting the four domains of CaV3.2 channels, allowing for rapid and precise modulation of channel function. Several protein kinases, including calmodulin-dependent protein kinase II (CaMKII), protein kinase A (PKA), and protein kinase C (PKC), are involved in this regulation. CaMKII primarily modulates T-type Ca^2+^ channel activation by enhancing their probability of opening through phosphorylation 84, 85. PKA and PKC similarly modulate Cav3.2 channels, typically by phosphorylating specific residues to enhance activity and thus increase Ca^2+^ influx and neuronal excitability, which contribute to pain signaling 86. Cyclin-dependent kinase 5 (Cdk5) is particularly notable for augmenting surface expression of Cav3.2 channels by targeting two specific residues, Ser561 and Ser1987 61. Increased Cdk5 levels in DRG after spinal nerve injury suggest a role in pain modulation. Pharmacological inhibition of Cdk5 with olomoucine has been shown to reduce compound action potentials and alleviate mechanical allodynia induced by spinal nerve ligation 61, 87. Ubiquitination is an important mechanism for regulating the stability, trafficking, and function of Cav3.2 channels. The ubiquitinating enzyme USP5, which specifically binds to the intracellular domain III-IV linker region of the channels, removes ubiquitin from these channels. USP5 expression is upregulated in DRG and spinal cord in inflammatory and neuropathic pain states 88, 89. Knockdown of USP5 via shRNA reduces Cav3.2 protein levels and attenuates Cav3.2 whole-cell currents 88, suggesting that the ubiquitinating activity of USP5 prevents degradation of Cav3.2 channels and maintains their functional presence at the cell surface. Disruption of the interaction between USP5 and Cav3.2 *in vivo* results in analgesic effects in both inflammatory and neuropathic pain models 88-91. Furthermore, SUMOylation of USP5 appears to decrease its affinity for Cav3.2 channels, indicating a complex regulatory interplay that influences pain signaling pathways.

PKC modulates the activity of various Cav channels through phosphorylation at specific serine and threonine residues primarily located on the intracellular loops and C-terminal domains of the α1 subunit. In L-type channels, Ser1928 on Cav1.2 is a well-characterized PKC target that modulates channel activity in various tissues 92. For N-type and P/Q-type channels, PKC enhances channel activity by phosphorylation of the α1 subunit, particularly at the domain I-II intracellular linker, which contributes to increased channel opening at presynaptic terminals 93. Additionally, PKC targets the synprint site (a region critical for SNARE protein binding) at Ser774 and Ser898 of N-type channels, further modulating synaptic transmission 94. In T-type channels, phosphorylation of Cav3.2 channels by Cdk5 and PKC has been linked to enhanced channel activity in neuropathic pain models 95, 96, suggesting a role for these kinases in pain sensitization.

### Transient Receptor Potential (TRP) channels

Transient receptor potential (TRP) channels form a large family of ion channels that play a key role in various sensory processes, including pain perception 97. These channels act as polymodal detectors that respond to a variety of physical and chemical stimuli. The TRP channel superfamily is divided into six subfamilies: TRPC (Canonical), TRPV (Vanilloid), TRPM (Melastatin), TRPA (Ankyrin), TRPP (Polycystin), and TRPML (Mucolipin). Among these subfamilies, TRPV1 and TRPA1 channels, which are also Ca^2+^-permeable channels, are particularly important in pain pathways (Figure 1). These two channels are extensively involved in nociception and play a key role in acute and chronic pain states. Their ability to respond to a wide range of noxious stimuli and their involvement in various pain modalities make them critical components of the pain signaling network and important targets for pain research and therapeutic development.

#### TRPV1

TRPV1 is a polymodal receptor activated by noxious heat, capsaicin, acidic pH, and endogenous ligands, which are often associated with inflammation. It is primarily expressed in nociceptors associated with unmyelinated C fibers and medium-diameter thinly myelinated Aδ fibers. When activated, TRPV1 promotes the influx of cations (Ca^2+^ and Na^+^) into the nociceptive neuron, causing depolarization and initiation of action potentials. This channel is involved in both peripheral and central sensitization processes, contributing to conditions such as thermal hyperalgesia and inflammatory pain 98, 99.

TRPV1 regulation in the pain pathways involves multiple intricate mechanisms. During inflammation, TRPV1 undergoes posttranslational modifications that significantly increase its sensitivity to activation stimuli. This process is mediated by the local release of various inflammatory mediators, such as substance P, CGRP, bradykinin, cytokines (e.g., TNF-α, IL-1β), nerve growth factor (NGF), and other factors 100. These inflammatory agents sensitize TRPV1 by lowering its activation threshold and/or increasing its expression. Phosphorylation by kinases such as PKA, PKC, and Src often enhances TRPV1 sensitivity through various mechanisms, including increased channel activity, reduced desensitization, and increased membrane trafficking 101. PKC-mediated phosphorylation of TRPV1 occurs at Ser 502, Tyr704, and Ser 800 102. Phosphorylation of Ser502 contributes to capsaicin-induced hypersensitivity following PKC activation but does not affect responses to heat or acid. Tyr704 is selectively involved in heat hypersensitivity. In contrast, Ser800 serves as a polymodal sensitization site, integrating multiple inflammatory signals in nociceptors and contributing to hypersensitivity across capsaicin, heat, and acid stimuli. Functional studies have shown that mutations at these sites abolish PKC-induced sensitization, underscoring their critical role in regulating TRPV1 activity 103. Lipid regulation, particularly by phosphoinositides and endocannabinoids, can either sensitize or inhibit TRPV1 activity 104-106. The function of the channel is further influenced by interactions with proteins such as calmodulin 107 and AKAP79/150 108. Environmental factors such as increased temperature 109 and acidic pH 110, common in inflamed tissues, directly activate TRPV1. This multifaceted modulation of TRPV1 highlights its central role in pain perception and its potential as a target for pain management strategies. Because of its central role in the pain pathway, TRPV1 is an important target for analgesic development, with both antagonists and agonists being explored for pain management. Although preclinical studies have established a critical role for TRPV1 in various pain conditions, clinical trials have encountered challenges, one of which, the induction of hyperthermia, has been a major issue 111.

#### TRPA1

TRPA1 is an important TRP channel in pain pathways, activated by a diverse range of chemical, noxious cold, and mechanical stimuli. TRPA1 is specialized for sensing endogenous disease-causing agents such as reactive oxygen species (ROS), reactive nitrogen species (RNS), and inflammatory mediators such as bradykinin and cytokines. These agents sensitize TRPA1 channels, thereby increasing nociceptor hypersensitivity and contributing to pain perception. TRPA1 is expressed on nociceptive neurons in peripheral tissues, where it detects harmful stimuli and contributes to the initiation of pain signals, especially in inflammatory conditions 112. TRPA1 is also present on the central terminals of nociceptive neurons in the spinal dorsal horn, where it amplifies pain transmission and contributes to central sensitization 113. When TRPA1 is activated, neuropeptides, including substance P and CGRP that mediate neurogenic inflammation and further sensitize nociceptors, are released. In neuropathic pain models, TRPA1 activation following nerve injury contributes to mechanical allodynia and cold hypersensitivity 114, 115, and in Schwann cells orchestrates neuroinflammation, ethanol and oxidative stress, sustaining neuropathic pain 116, 117. During inflammation, TRPA1 activation by inflammatory agents leads to nociceptor hypersensitivity via pathways such as PLC/Ca^2+^ signaling 112. This channel has also been implicated in cancer pain, osteoarthritis, chemotherapy-induced neuropathy, migraine, and postoperative pain 112. In these conditions, TRPA1 activation or blockade increases or decreases pain behavior and hypersensitivity accordingly. The wide-ranging activation profile of TRPA1 and its role in diverse pain pathways make it an attractive target for the development of analgesic drugs aimed at treating various types of pain, including inflammatory, neuropathic, and migraine pain.

While TPRA1 shares several regulatory mechanisms with TRPV1, including phosphorylation by PKA and PKC, lipid regulation, and interactions with intracellular proteins, it also has unique regulatory features. Specific phosphorylation sites on TRPA1 have been identified that are important for its regulation by PKC. Notably, phosphorylation at Ser119, Thr281, and Thr529 play key roles in sensitizing the channel 118. In addition to functioning as a pain sensor, TRPA1 also functions as a sensor of oxidative stress. It can be directly activated by ROS and RNS through oxidation of specific cysteine residues 119. A unique characteristic of TRPA1 is its activation by covalent modifications, particularly by electrophilic agents such as mustard oil, which directly modify cysteine ​​residues on the channel 120. These distinct regulatory mechanisms highlight the special role of TRPA1 in sensing not only painful stimuli but also cellular stress and specific chemical agents, contributing to its importance in various pain and inflammatory conditions.

### Calcium-Activated Ion Channels in Pain Pathways

In addition to VGCCs and TRP channels, calcium-activated ion channels play a significant role in the onset and maintenance of pain by modulating neuronal excitability and synaptic transmission in pain pathways. These channels are activated by intracellular Ca²⁺, often downstream of Ca^2+^ influx through VGCCs or TRP channels, and include calcium-dependent chloride channels, such as anoctamin 1 (ANO1), and calcium-dependent potassium channels, such as large-conductance (BK_Ca_) and small-conductance (SK_Ca_) channels.

#### Anoctamin 1 (ANO1)

ANO1, alternatively known as TMEM16A, is a calcium-activated chloride channel widely expressed in nociceptive DRG neurons and dorsal horn neurons 121-123. ANO1 is functionally coupled with TRPV1 channels. When calcium enters through TRPV1, it triggers ANO1 activation, resulting in chloride efflux that depolarizes neurons and heightens their excitability 124, 125. ANO1 enhances neuronal excitability in inflammatory and neuropathic pain, contributing to hyperalgesia, and pharmacological or genetic inhibition of ANO1 in nociceptive neurons attenuates pain behaviors 121, 122.

#### BK_Ca_ (large-conductance)

Large-conductance calcium-activated potassium (BK_Ca_) channels, encoded by the *KCNMA1* gene, are expressed in DRG neurons and spinal cord neurons and are activated by both Ca^2+^ and membrane depolarization 126. BK_Ca_ channels contribute to the repolarization phase of action potentials and mediate the afterhyperpolarization (AHP), which regulates the firing frequency of neurons. In the context of pain, BK_Ca_ channels generally play an antinociceptive role by reducing neuronal excitability. BK_Ca_ channel activity is downregulated in DRG neurons following peripheral injury, leading to increased excitability and contributing to inflammatory or neuropathic pain 127-129. Intrathecal delivery of viral vectors or pharmacological activation of BK_Ca_ channels with agonists like NS1619 reduces excitability in nociceptive neurons and attenuates mechanical and thermal hyperalgesia in animal models of neuropathic and inflammatory pain 127, 129-131. Conversely, inhibition of BK_Ca_ channels with blockers like iberiotoxin enhances neuronal firing and exacerbates pain behaviors, underscoring their protective role 127, 132.

#### SK_Ca_ (small-conductance)

Small-conductance calcium-activated potassium (SK_Ca_) channels, encoded by *KCNN1-3* genes, are expressed in both peripheral nociceptors and spinal cord neurons and are activated solely by Ca^2+^ binding to calmodulin 133. SK_Ca_ channels contribute to the medium AHP, which regulates spike frequency adaptation and neuronal excitability 134. Silencing SK channels in Drosophila results in heightened pain sensitivity behaviors, suggesting these channels function to attenuate pain signaling pathways 135. In injured human and mouse DRG, KCNN1(SK1) expression is downregulated, leading to increased DRG neuron excitability and contributing to the development of neuropathic pain 136-139. Restoring KCNN1 expression in injured DRG, either through sensory neuron-specific long non-coding RNA modulation 136 or viral-mediated gene delivery 137, effectively reduces neuronal hyperexcitability and attenuates neuropathic pain behaviors in animal models.

## Ca^2+^ Imaging in Pain Research

*In vivo* or *ex vivo* Ca^2+^ imaging has emerged as a powerful technique in pain research, offering significant advantages over traditional cultured neuron imaging. These methods employ Ca^2+^-sensitive dyes or other Ca^2+^ indicators, most typically, genetically encoded Ca^2+^ indicators (GECIs), to visualize and measure Ca^2+^ dynamics in neurons and other cells involved in the pain pathway. Key advantages of these techniques include the preservation of neurons in their natural context, the maintenance of native connectivity and microenvironmental factors; the capture of complex interactions between neurons and non-neuronal cells in intact networks; the observation of dynamic processes such as real-time responses to stimuli and pharmacological effects; and the capacity to study whole-system responses in mature neurons within intact tissues. These benefits provide a comprehensive and physiologically relevant view of neuronal function that is critical for understanding the intricate mechanisms underlying pain perception and processing.

### Ca^2+^ indicators

#### Ca^2+^-sensitive dyes

Ca²⁺-sensitive dyes are designed to selectively bind to intracellular Ca²⁺ ions, causing a conformational change that alters the fluorescence properties of the dye. This change in fluorescence intensity correlates with the concentration of Ca^2+^ ions, allowing visualization and measurement of real-time Ca^2+^ dynamics. Ca²⁺-sensitive dyes are less favored for *in vivo* Ca^2+^ imaging because their use involves invasive procedures, as they require injection to introduce the dye into the target tissue. These procedures lack cell type specificity, involve time-consuming loading procedures, and are of limited suitability for long-term studies 140.

#### GECI

GECIs are generally preferred over Ca²⁺-sensitive dyes as they offer targeted expression, stable long-term imaging, and a less invasive introduction method. GECIs are typically based on fluorescent proteins linked to Ca^2+^ binding domains, such as calmodulin or troponin C. Upon binding to Ca^2+^, these proteins undergo a conformational change that either enhances or quenches fluorescence. This change in fluorescence intensity correlates with Ca^2+^ concentration, providing a proxy for neuronal activity 141.

GCaMPs were first introduced in 2001 and have become the most widely used Ca^2+^ probes for studying *in vivo* Ca^2+^ activity. These GECIs are based on circular permutations of GFP fused to calmodulin and the M13 peptide from myosin light chain 142. The design of GCaMPs allows them to emit fluorescence in response to Ca^2+^ binding, providing a real-time readout of intracellular Ca^2+^ dynamics. Over the years, various GCaMP variants have been developed, offering different sensitivities and kinetics, applicable to diverse experimental needs. Early versions, GCaMP1 and GCaMP2, served as proof-of-concept but had limitations in sensitivity and dynamic range 142-145. GCaMP3 showed significant improvements, offering better signal-to-noise ratio and enhanced sensitivity 8, 9. The GCaMP5 series introduced various subtypes, such as GCaMP5A, GCaMP5G, and GCaMP5K, each optimized for different experimental conditions including kinetics and brightness as well as species compatibility 146. The GCaMP6 series includes GCaMP6s (slow), GCaMP6m (medium), and GCaMP6f (fast), providing options for different signal kinetics and amplitudes 147. The latest generation, jGCaMP8, offers improved performance characteristics including improved sensitivity and reduced photobleaching, making it the most advanced option for Ca^2+^ imaging to date 148. Each generation of GCaMPs was built on the strengths of the previous generation and addresses its limitations, making these indicators invaluable tools for exploring Ca^2+^ signaling in various biological contexts, including pain research.

**Figure 2:**
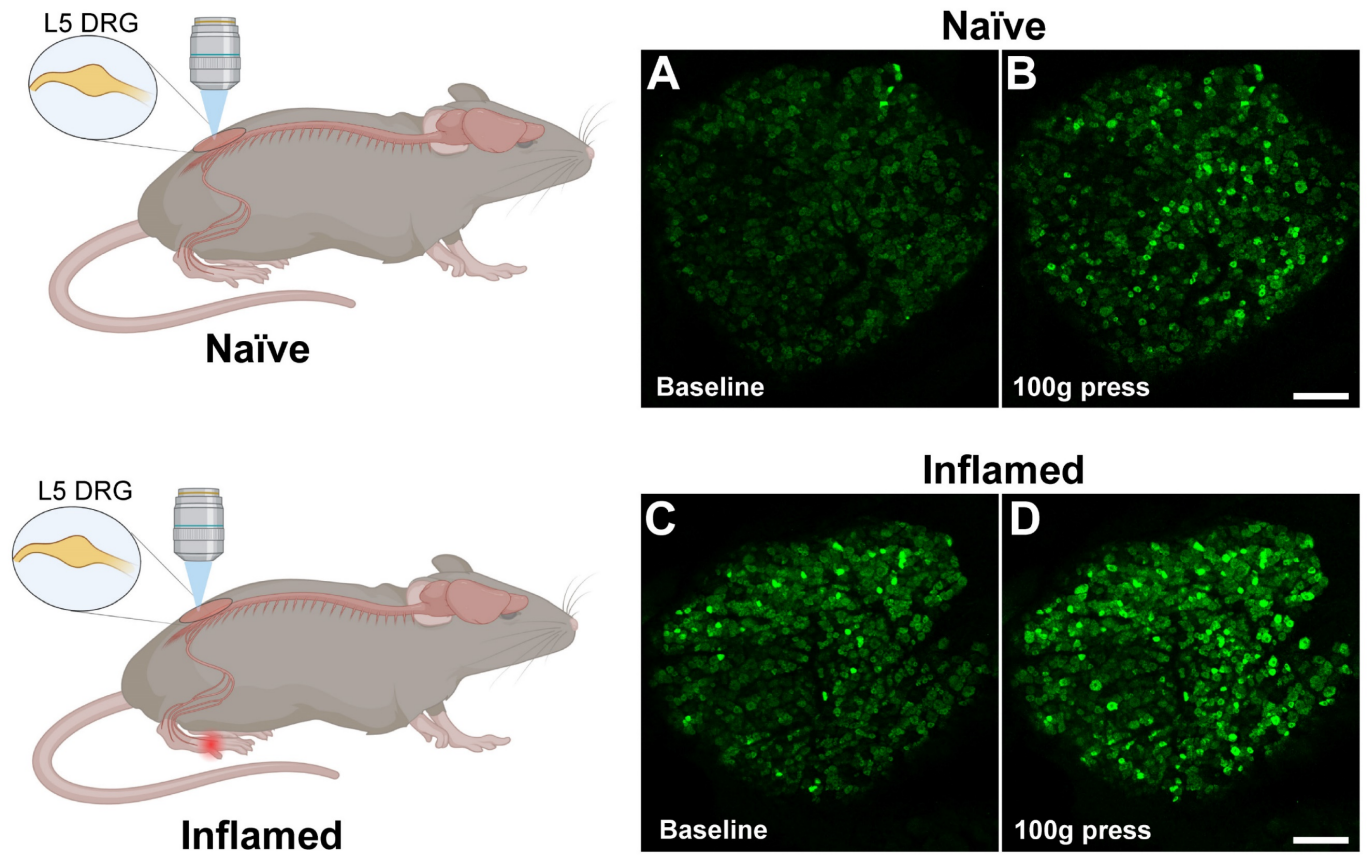
*In vivo* fluorescence imaging of dorsal root ganglion (DRG) during inflammation using Pirt-GCaMP3 mice. **A.** (*top*) Control mice received mock injection in right hindpaw; (*bottom*) Experimental mice received injection of complete Freund's adjuvant (CFA) into the right hindpaw to induce inflammation. For both mice, two days later, the DRG was surgically exposed at the right lumbar L5, which innervates the right hindpaw and examined by Ca^2+^ imaging. **B.** Representative confocal fluorescence images of the L5 DRG showing GCaMP3 expression in primary sensory neurons (green). At baseline, naïve mouse (*panel a*) showed few steady-state high Ca^2+^ level cells and Ca^2+^oscillated cells (spontaneous activity), whereas CFA-treated mouse (*panel c*) exhibited some steady-state high Ca^2+^ level cells and Ca^2+^oscillated cells (indicated by greener and brighter neurons). Among mice that received a 100-g mechanical press delivered to the right hindpaw (*panel b, d*), there was an increase in the number of activated DRG neurons (indicated by greener and brighter neurons), and the increase was much greater in the CFA-treated mice than in the control mice (compare *panels b & d*). Scale bar: 100 μm.

Despite the availability of other GECIs, such as Förster resonance energy transfer (FRET)-based GECIs like Twitch 149, which rely on energy transfer between two fluorescent proteins to report calcium levels, and red fluorescent GECIs like jRGECO1a 150 and RCaMP 151, which emit red fluorescence in response to calcium binding, GCaMP still remains the primary choice for *in vivo* Ca^2+^ imaging in pain studies. This preference is a consequence of several key advantages: GCaMPs provide superior signal strength and sensitivity, resulting in a higher signal-to-noise ratio that allows detection of subtle Ca^2+^ transients. They have a broad dynamic range allowing them to capture both small and large Ca^2+^ fluctuations, which is important for accurately monitoring pain-related neural activity. GCaMPs have become the preferred choice for Ca^2+^ imaging in pain research, in part, because the continuous optimization of GCaMPs has resulted in variants with improved performance characteristics such as faster kinetics and reduced baseline fluorescence. The increasing popularity of cell type-specific GCaMPs, such as those specifically expressed in nociceptors (Figure 2), has provided deeper insights into the molecular and cellular mechanisms underlying various pain conditions. The user-friendly nature, broad compatibility, and extensive validation of GCaMPs have established them as reliable and versatile tools, resulting in widespread adoption and a robust foundation of protocols and comparative data in the field.

### Applications in pain research

Unlike *in vitro* studies, *in vivo* imaging preserves the integrity of the cellular and tissue microenvironment, including the complex interactions between neuronal and non-neuronal cells. This preservation is essential for studying pain mechanisms, as it allows for a more accurate representation of how these cells communicate and function within their native context. In contrast, *ex vivo* imaging provides a more controlled setting for investigating pharmacological effects, especially when using perfusion techniques. In *ex vivo* setups, tissues can be exposed to precise concentrations of drug, ensuring consistent and uniform exposure while allowing direct observation of tissue responses without the confounding influence of the animal's physiological state. Thus, *ex vivo* imaging is particularly valuable for assessing the pharmacological effects of calcium channel blockers. By combining a variety of Ca^2+^ markers, *in vivo* and *ex vivo* imaging techniques have greatly improved our understanding of how different calcium channel subtypes affect neuronal excitability and synaptic transmission in the pain pathway, as summarized in Table 1.

Although many types of calcium channels are involved in pain transmission, studies of only a few have used *in vivo* Ca^2+^ imaging, and the *in vivo* regulatory mechanisms of most types remain largely unexplored. While *in vivo* Ca^2+^ imaging has provided important insights into many aspects of neuronal function, its application has been relatively constrained due to several technical challenges. (1) Achieving high spatial resolution with *in vivo* Ca^2+^ imaging can be challenging, especially in deep brain regions or densely packed areas like the spinal cord. This limitation hinders the precise localization of calcium channel activity within neurons and non-neuronal cells involved in pain pathways. (2) *In vivo* imaging is highly susceptible to motion artifacts due to freely moving animals or physiological processes such as breathing and heartbeat. These artifacts can complicate Ca^2+^ signal analysis and prevent accurate observations. (3) *In vivo* Ca^2+^ imaging typically reflects the overall Ca^2+^ dynamics within a cell or tissue. This makes it challenging to isolate the activity of specific Ca²⁺ channel subtypes involved in pain pathways, especially when multiple types of Ca²⁺ channels contribute to the observed Ca²⁺ transients. (4) Ca^2+^ imaging relies on changes in intracellular Ca^2+^ levels as a proxy for neuronal activity, but these changes are slower and more indirect than membrane potential changes captured by voltage imaging. Voltage imaging using genetically encoded voltage indicators (GEVIs) directly measures membrane potential fluctuations with millisecond precision, capturing fast electrical events like action potentials and subthreshold depolarizations that are critical in pain signaling 160. This makes voltage imaging better suited for studying the temporarily precise timing and dynamics of nociceptive signaling, particularly in fast-spiking neurons involved in pain responses.

These challenges highlight the need for further technological advances and innovative approaches to overcome these limitations if we are to gain a comprehensive understanding of calcium channel regulation in pain pathways. Future developments in imaging technologies and genetic tools may help address these issues and may potentially lead to more precise and informative studies of calcium channel regulation in pain processing *in vivo*.

### Future directions

The use of genetically modified animals (e.g., transgenic mice or mice with specific gene silencing) in combination with *in vivo* calcium imaging will provide a powerful approach for elucidating the role of specific calcium channel subtypes in pain pathways. This method will provide detailed insights into how genetic alterations affect neural activity and pain signaling within living animals. By applying this technique across various pain models, including inflammatory, neuropathic, and other chronic pain models, as well as disease-associated transgenic mutant mice, we can unravel the complex mechanisms underlying diverse pain states and elucidate the specific contributions of different calcium channel subtypes. This approach has the potential to identify new targets for pain management and to deepen our understanding of the molecular and cellular basis of pain perception and modulation.

## Conclusion

Ca^2+^ and Ca^2+^ channels are fundamental to the regulation of pain pathways, with different subtypes contributing to different aspects of pain perception and modulation. Advances in *in vivo* Ca^2+^ imaging, particularly using GECIs such as GCaMP, have greatly improved our understanding of the neural and non-neuronal dynamics in pain processing. However, a notable gap persists in the field with respect to using *in vivo* Ca^2+^ imaging to fully elucidate the regulatory role of specific Ca^2+^ channel subtypes in pain pathways. Current research has yet to fully explore the complex interactions and precise roles of different Ca^2+^ channel subtypes *in vivo*, and bridging this gap will be critical to developing novel therapeutic approaches targeting specific Ca^2+^ channels, which may lead to more effective pain relief strategies, particularly for chronic pain conditions. Progress in this direction holds the promise of unveiling new insights into pain mechanisms and opening avenues for innovative pain management techniques.

## Figures and Tables

**Table 1:** Current *in vivo* and *ex vivo* imaging techniques use in studying the regulatory mechanisms of Ca^2+^ channels in pain transmission

Ca^2+^ indicator	Species	Imaging method	Experimental approach	Regulatory mechanism(s)	Year
GCaMP6s	Transgenic mice (Trpv1-GaMP6s)	*Ex vivo* 2-photon imaging of L4 DRG neuron central terminals	Pharmacological blockage	N- and P/Q-type channels mediate the increased Ca^2+^ amplitude in central terminals of DRG neurons following spared nerve injury.	2024 152
GCaMP6f	Transgenic mice (Advillin-GaMP6f)	*Ex vivo* imaging of spinal cord slices	Genetic ablation	TRPA1 is essential for the Ca^2+^ response in axonal terminals triggered by microRNA lethal-7 (let-7), which acts as a potent pain inducer.	2024 153
GCaMP6m	Mice (AAV expression)	*In vivo* optical fiber photometer imaging of brain	Pharmacological blockage	The functional upregulation of T-type Ca^2+^ channels induced by remifentanil increases activity in the thalamocortical VPL^Glu^→S1HL^Glu^ circuit, contributing to the development of secondary pain in mice.	2022 154
GCaMP3	Transgenic mice (Pirt-GCaMP3)	*In vivo* confocal imaging of trigeminal ganglia	Pharmacological blockage	Stress and/or pituitary adenylate cyclase-activating polypeptide-38 (PACAP38) elevation leads to nociceptor sensitization via TRPV1.	2024 155
GCaMP3	Transgenic mice (Pirt-GCaMP3)	*In vivo* confocal imaging of dorsal root ganglia	Agonist pretreatment	Desensitization of TRPV1 in nociceptors alleviate postoperative surgery pain by reducing neuronal hyperexcitability.	2021 156
GCaMP3	Transgenic mice (Pirt-GCaMP3)	*In vivo* confocal imaging of trigeminal ganglia	shRNA interference	Upregulated descending serotonergic input causes central terminal TRPV1 sensitization in the dorsal horn and contributes to chronic neuropathic pain.	2014 8
GCaMP	Transgenic C. elegans	*In vivo* imaging of ASH neurons	Diet supplementation	Phosphoinositide lipids are negative modulators of TRPV1 *in vivo*.	2021 157
FRET	Transgenic C. elegans	*In vivo* imaging of thermonociceptor	mutations	(1) N-type channels play a crucial role in thermal sensitivity. (2) L-type channels help maintain high sensory gain. (3) Neither channel type is essential for sustaining stable Ca^2+^ signaling during long-term stimulation.	2019 158
Ca^2+^-sensitive dye	Mice (dye injection)	*In vivo* 2-photon imaging of spinal cord	Genetic ablation	(1) TRPM8-positive DRG neurons are responsible for spinal responses to mild cooling. (2) TRPV1-positive DRG neurons are responsible for spinal responses to heat and strong cold.	2016 159
